# Serum CXCL12 and S100A12 levels in peripheral blood fluid and their correlation with severity in patients with knee osteoarthritis

**DOI:** 10.5937/jomb0-46310

**Published:** 2025-01-24

**Authors:** LijiangTao, He Minjuan, Lu Yisheng, Zheng Jie, Ye Yili

**Affiliations:** 1 Zhejiang Provincial People's Hospital, Department of Orthopedics, Hangzhou, China; 2 The 903 Hospital of the Joint Service Support Department of the Chinese People's Liberation Army, Department of Orthopedics, Hangzhou, China; 3 Taizhou Traditional Chinese Medicine Hospital (Currently in the Logistics Support Department), Department of Orthopedics, Taizhou, China

**Keywords:** knee osteoarthritis, CXCL12, S100A12, correlation, osteoartritis kolena, CXCL12, S100A12, korelacija

## Abstract

**Background:**

This paper aimed to investigate the expression of CXCL12 and S100A12 in peripheral blood (PB) and synovial fluid (SF) of patients with knee osteoarthritis (OS) and to analyze the correlation between them and the severity of knee OS.

**Methods:**

Sixty patients with knee OS treated in our hospital from January 2020 to December 2022 were selected as the experimental group, and 60 healthy knee joints with similar ages were selected as the control group. The fasting venous blood of 120 subjects was drawn in the early morning, and the SF was extracted during joint operation or sodium hyaluronate injection. Put the collected PB and SF in the refrigerator at -80 °C. The levels of CXCL12 and S100A12 in PB and SF were detected by enzyme-linked immunosorbent assay (Elisa).

**Results:**

The correlation between the levels of CXCL12 and S100A12 in PB and SF and Kmurl L grade and WOMAC score. The levels of CXCL12 and S100A12 in PB and SF in the observation group were higher than those in the control group. There were significant differences in the levels of CXCL12 and S100A12 in PB and SF in the experimental group. The higher the Kmurl grade of knee OS, the higher the concentration of CXCL12 and S100A12 in PB and SF. The levels of CXCL12 and S100A12 in PB of knee OS were positively correlated with WOMAC score (r=0.767, 0.521, respectively, P<0.05); see Figure 1. The levels of CXCL12 and S100A12 in SF of knee OS were positively correlated with WOMAC score (r=0.663, 0.357 respectively, P<0.05). The levels of CXCL12 and S100A12 in PB and SF are positively correlated with the severity of knee OS.

**Conclusions:**

The levels of CXCL12 and S100A12 in PB and SF can provide the basis for the evaluation and prognosis of knee OS.

## Abstract (sr)

**Uvod:**

Ovaj rad je imao za cilj da istraži ekspresiju CXCL12 i S100A12 u perifernoj krvi (PB) i sinovijalnoj tečnosti (SF) kod pacijenata sa osteoartritisom kolena (OS) i da analizira korelaciju između njih i težine osteoartritisa kolena.

**Metode:**

Kao eksperimentalna grupa odabrano je 60 pacijenata sa osteoartritisom kolena koji su lečeni u našoj bolnici od januara 2020. do decembra 2022. godine, dok je kao kontrolna grupa odabrano 60 zdravih osoba sličnih godina sa zdravim kolenima. U ranim jutarnjim časovima uzeta je venska krv na prazan stomak od 120 ispitanika, a SF je izvučena tokom operacije zgloba ili injekcije natrijumhijaluronata. Sakupljeni PB i SF su stavljeni u frižider na -80 °C. Nivoi CXCL12 i S100A12 u PB i Sf su detektovani pomoću enzimski povezane imunosorbentne analize (Elisa).

**Rezultati:**

Korelacija između nivoa CXCL12 i S100A12 u perifernoj krvi (PB) i sinovijalnoj tečnosti (SF) sa Kmurl L klasifikacijom i WOMAC skorom. Nivoi CXCL12 i S100A12 u PB i SF u posmatranoj grupi bili su viši nego u kontrolnoj grupi. Postojale su značajne razlike u nivoima CXCL12 i S100A12 u PB i SF u eksperimentalnoj grupi. to je veći Kmurl stepen osteoartritisa kolena, to je veča koncentracija CXCL12 i S100A12 u PB i SF. Nivoi CXCL12 i S100A12 u PB kod osteoartritisa kolena su bili u pozitivnoj korelaciji sa WOMAC skorom (r=0,767, 0,521, respektivno, P<0,05) - vidi Sliku 1. Nivoi CXCL12 i S100A12 u SF kod osteoartritisa kolena su bili u pozitivnoj korelaciji sa WOMAC skorom (r=0,663, 0,357 respektivno, P<0,05). Nivoi CXCL12 i S100A12 u PB i SF su bili u pozitivnoj korelaciji sa ozbiljnošću osteoartritisa kolena.

**Zaključak:**

Nivoi CXCL12 i S100A12 u PB i SF mogu pružiti osnovu za evaluaciju i prognozu osteoartritisa kolena.

## Introduction

Osteoarthritis (OA) is a degenerative joint disease primarily affecting the knee joint. OA of the knee can cause swelling, pain, and impaired movement [1]. Despite significant progress in understanding the mechanisms of knee OA in recent years, treatments have lagged behind [2] [3]. Traditionally, the development of knee OA was predicted based on macromolecules or the external shape of the knee joint, which often resulted in low accuracy and potential misdiagnoses.

Recent advancements in etiology have shifted knee OA research to a molecular level. Studies have shown that inflammation is a key factor in the onset and progression of knee OA, involving elements such as chemokines, interleukins (IL), and calcium-binding proteins (CBP). For example, the continuous increase of chemokine 12 (CXCL12) in the human body may indicate inflammation. The level of CXCL12 correlates with the progression and prognosis of knee OA, but it is not yet clear whether the expression of CXCL12 in knee joint synovial fluid (SF) and peripheral blood (PB) is related [4] [5].

S100A12, a member of the S100 protein family, is another important factor secreted by synovial tissue and plays a crucial role in inflammatory mediation [6]. Therefore, this study analyzed the correlation between CXCL12, S100A12, and knee OA by detecting the expression levels in the knee joint.

## Materials and methods

### General information

Sixty patients with knee osteoarthritis (OA) treated at our hospital from January 2020 to December 2022 were selected as the experimental group. Selection criteria included: (1) meeting the diagnostic criteria for primary knee OA; (2) age between 50 and 68 years; (3) X-ray examination showing narrowed knee joint space and osteophyte formation at the edges; (4) presence of bone friction sounds in the knee joint. Exclusion criteria were: (1) presence of other joint infections; (2) patients who had intra-articular injections or external injuries within the past six months; (3) patients with autoimmune diseases; (4) patients who had used hormone medications. Sixty healthy individuals of similar age were selected as the control group.

### Collecting method

Peripheral blood (PB) extraction method: Both groups fasted for 12 hours prior to the blood draw. Fasting venous blood was collected at 6:30 AM. After collection, the supernatant was separated into a vacuum tube and centrifuged immediately.

Joint fluid extraction methods included (1) extraction during knee surgery and (2) extraction during joint puncture followed by the injection of sodium hyaluronate. After collecting the supernatant and synovial fluid (SF), samples were stored in a refrigerator at -80°C.

### Observation index

Sixty patients with knee osteoarthritis (OA) were divided into four groups according to the Kellgren-Lawrence (KL) scale’s standard grading scale. The higher the grade, the more severe the lesion, with a grade of 0 indicating a healthy condition. Therefore, the control group was classified as grade 0 on the KL scale. Patients in the control group also had their knee joints assessed using the WOMAC OA index score [7] [8]. A higher WOMAC score indicates a greater degree of knee OA.

CXCL12 levels were detected using enzyme-linked immunosorbent assay (ELISA), with the test kit purchased from Ruisai Biotechnology Co., Ltd. S100A12 levels were also determined by ELISA, using a kit purchased from Shenzhen Aikang Electronics Co., Ltd. All reagents were used in strict accordance with the product instructions.

### Statistical method

SPSS20.0 statistical software was used, and the measurement data were expressed as (x̄±s). An independent sample t-test was used to compare the two measurement data groups, and the Kmuri W (Kruskal-Wallis) test was used to compare multiple data groups. CXCL12 and S100A12 were analyzed using regression analysis, Spearman correlation analysis, and the correlation between Kmurl L grade and WOMAC score. P<0.05 has statistical significance.

## Results

### General characteristics of included patients

There were 60 cases in the experimental group, including 28 males and 32 females, aged (63.31±8.14) years. In the control group, there were 24 males and 36 females, with an age of (55.26±15.28) years. There was no significant difference in sex and age between the two groups (P>0.05). In the experimental group, 60 patients were graded by Kellgren-Lawerence (Kmurl) scale [7] by X-ray fluoroscopy, which was divided into grade I–IV. Among the 60 patients in the experimental group, there were 17 cases of grade I, 15 cases of grade II, 18 cases of grade III and 10 cases of grade IV. Table 1

**Table 1:** General characteristics of the included population.

Characteristic	Experimental Group<br>N=60	Control Group<br>N=60	P-value
Gender			
Male	28 (46.7%)	24 (40.0%)	0.539
Female	32 (53.3%)	36 (60.0%)	
Age (years)	63.31±8.14	55.26±15.28	0.001
Grade			
I	17 (28.3%)	-	-
II	15 (25.0%)		
III	18 (30.0%)		
IV	10 (16.7%)		

### The levels of CXCL12 and S100A12

The study measured the levels of CXCL12 and S100A12 in peripheral blood (PB) and synovial fluid (SF) in both the experimental and control groups. The mean PB CXCL12 level in the experimental group was 7.35±1.81 ng/mL, significantly higher than the control group’s mean of 2.75±1.23 ng/mL (P<0.05). Similarly, the SF CXCL12 level in the experimental group was 8.32±1.49 ng/mL, compared to 3.13±1.64 ng/mL in the control group (P<0.05). For PB S100A12, the experimental group had a mean level of 35.10±26.43 ng/mL, significantly higher than the control group’s 22.13±15.67 ng/mL (P<0.05). Additionally, SF S100A12 levels were 14.09±8.08 ng/mL in the experimental group, compared to 9.30±6.85 ng/mL in the control group (P<0.05). These results indicate that both CXCL12 and S100A12 levels are significantly elevated in the experimental group, suggesting a potential association of these biomarkers with the condition under study. Table 2

**Table 2:** General characteristics of the included population.

Group	n	PB CXCL12 (ng/mL)	SF CXCL12 (ng/mL)	PB S100A12 (ng/mL)	SF S100A12 (ng/mL)
experimental	60	7.35±1.81	8.32±1.49	35.10±26.43	14.09±8.08
control	60	2.75±1.23	3.13±1.64	22.13±15.67	9.30±6.85
*P*		0.05	0.05	<0.05	<0.05

### Differences of CXCL12 in PB and SF in different KL grades

In the experimental group, the higher the Kmurl grade of knee OS, the higher the concentration of CXCL12 in PB and arthritis (Table 3). The differences in CXCL12 levels across the KL grades were statistically significant, with P<0.01 for both PB and SF measurements. These findings indicate a progressive increase in CXCL12 levels in both PB and SF corresponding to the severity of the KL grades, suggesting that higher CXCL12 levels are associated with more advanced stages of the condition.

**Table 3:** Differences of CXCL12 levels in PB and synovial fluid among different Kmurl grades (x̄±s).

KL grades	n	PB blood CXCL12 (ng/mL)	SFCXCL12 (ng/mL)
I	17	4.56±1.21	4.89±1.33
II	15	6.55±0.69	6.86±1.89
III	18	8.96±1.97	8.36±1.79
IV	10	10.49±1.25	11.62±2.47
*P*		<0.01	<0.01

### Difference of S100A12 in PB and SF in different KL grades

In the experimental group, the higher the Kmurl grade of knee OS, the higher the concentration of S100A12 in PB and arthritis (Table 4). The differences in CXCL12 levels among the KL grades were statistically significant (P<0.01), indicating an increase in CXCL12 levels corresponding to the severity of the KL grades.

**Table 4:** Difference of S100A12 in PB and SF with different Kmurl grades.

KL grades	n	PBS100A12 (ng/mL)	SFS100A12 (ng/mL)
I	17	7.35±1.25	4.55±1.23
II	15	9.42±2.31	6.25±3.55
III	18	22.36±5.26	9.58±6.23
IV	10	46.21±14.56	17.59±4.89
*P*		<0.01	<0.01

These differences in S100A12 levels were also statistically significant (P<0.01), demonstrating a substantial increase in S100A12 levels in both PB and SF with the progression of KL grades. The data suggest that higher levels of CXCL12 and S100A12 are associated with more advanced stages of the condition, highlighting their potential role as biomarkers for disease severity.

### Correlation between CXCL12 and S100A12 levels in PB and SF and WOMAC score

The levels of CXCL12 and S100A12 in PB of knee OS were positively correlated with WOMAC score (r=0.767 and 0.521, respectively, P<0.05). The levels of CXCL12 and S100A12 in knee OSSF were positively correlated with the WOMAC score (r=0.663 and 0.357, P<0.05), as shown in Table 5.

**Table 5:** Correlation between CXCL12 and S100A12 levels in PB and SF and WOMAC score.

	r	P
PBCXCL12	0.767	<0.05
SFCXCL12	0.663	<0.05
PBS100A12	0.521	<0.05
SFS100A12	0.357	<0.05

## Discussion

From an anatomical perspective, the knee joint can be divided into cartilage, subchondral bone, and synovium. Inflammation in the knee joint arises from excessive friction between these tissues in an unstable joint. Therefore, osteoarthritis (OA) of the knee joint is caused by instability in multiple joint tissues [9]. The development of knee OA is not solely due to tissue friction but also involves the participation of various inflammatory factors, which play a crucial role in inducing the condition.

In modern medicine, knee OA’s diagnosis and severity evaluation primarily depends on patient self-reports and imaging data. However, this approach has limitations, particularly for patients whose bone and joint structure and shape have not yet changed and whose early symptoms are not obvious [10] [11]. Consequently, more accurate indicators are needed to diagnose and evaluate knee OA. Inflammatory factors present in knee OA can be detected in blood and synovial fluid (SF), offering potential for early diagnosis, disease progression monitoring, and prognosis assessment.

Studies have shown that chemokines are involved in developing knee OA, with CXCL12 playing a significant role in inflammation and immune dysfunction associated with knee OA. CXCL12 stimulates the release of inflammatory factors, exacerbating inflammation and increasing the inflammatory burden on the knee joint. Additionally, CXCL12 can promote leukocyte chemotaxis, increasing leukocyte levels in the blood and enhancing the inflammatory response. By directly amplifying the inflammatory response, CXCL12 can worsen the patient’s condition [12] [13].

CXCL12 also contributes to the degeneration of articular cartilage and the apoptosis of chondrocytes, leading to increased friction between joint tissues and further aggravating inflammation. S100A12, another pro-inflammatory factor, facilitates the migration of neutrophils to inflammatory sites in the knee joint, increasing the inflammatory burden. Neutrophils play a strong chemotactic role, promoting the progression of inflammation [14]. CXCL12 and S100A12 are highly sensitive biomarkers of inflammatory activity, allowing for the diagnosis, evaluation, and prognosis of inflammatory diseases by measuring their levels.

This study used the Kellgren-Lawrence (KL) grading method to classify knee OA into grades I to IV. The level of CXCL12 in the peripheral blood (PB) of the experimental group was significantly higher than that of the control group, indicating that CXCL12 is involved in joint inflammation in knee OA. The more severe the lesion, the higher the CXCL12 level, suggesting that CXCL12 can be used to evaluate the severity of knee OA. The level of S100A12 in PB of the experimental group was also significantly higher than that of the control group. S100A12, secreted by neutrophils and transported through the synovium into the synovial fluid (SF), encounters a synovial barrier that limits its transport, resulting in higher blood levels than in SF [15].

The level of CXCL12 in SF in the experimental group was significantly higher than in the control group, with high concentrations detected in the SF of patients with knee OA, increasing following the KL grade. CXCL12 levels correlate with the pathological severity of knee OA. The mechanism by which CXCL12 induces knee OA may involve the activation of the P38MAPK pathway, leading to the expression of MMP-13, COX-2, and other factors that cause biological changes in knee OA [16]. S100A12 levels in SF also increase with higher KL grades, indicating that S100A12 levels reflect the severity of knee joint disease and can predict the extent of knee joint injury.

Recent findings suggest that CXCL12 may trigger the expression or secretion of various MMPs. In osteoarthritis (OA), chondrocytes within the joint cartilage release elevated levels of MMPs, resulting in cartilage degradation [17]. Additionally, CXCL12 might indirectly contribute to joint cartilage degradation by increasing the synthesis of collagen type I [18]. A study proposed that CXCL12 originating from synoviocytes accumulates and becomes immobilized on heparan sulfate molecules of endothelial cells, potentially fostering angiogenesis and the infiltration of inflammatory cells [19].

Therefore, the results of this study suggest that measuring CXCL12 and S100A12 levels in PB and SF is crucial for evaluating the pathological changes in early knee OA. However, further research is needed to explore the early diagnosis and prevention of bone and joint diseases using S100A12 and CXCL12 and to conduct additional examinations such as pathology. This study has some limitations, including a small sample size, differences in autoimmunity, and age variations among participants. Thus, further research is necessary.

## Conclusion

The levels of S100A12 and CXCL12 in PB and SF were positively correlated with the Kmurl grade of knee OS. The levels of S100A12 and CXCL12 in PB and SF play a guiding role in early prevention, diagnosis, treatment and prognosis of OS. It can be used as an important index to analyze and evaluate the pathological degree of knee OS.

## Dodatak

### Conflict of interest statement

All the authors declare that they have no conflict of interest in this work.
